# iProDNet: integrated probabilistic differential network inference under heterogeneous biological conditions

**DOI:** 10.1093/bib/bbag565

**Published:** 2026-10-08

**Authors:** Heewon Park, Seiya Imoto

**Affiliations:** School of Mathematics, Statistics and Data Science, Sungshin Women’s University, 2, 34 dagil, Bomun-ro, Seongbuk-gu, Seoul, 02844, Republic of Korea; Data Science Center, Sungshin Women’s University, 2, 34 dagil, Bomun-ro, Seongbuk-gu, Seoul, 02844, Republic of Korea; M&D Data Science Center, Institute of Integrated Research, Institute of Science Tokyo, 1-5-45 Yushima, Bunkyo-ku, Tokyo 113-8510, Japan; Human Genome Center, Institute of Medical Science, University of Tokyo, 4-6-1 Shirokanedai, Minato-ku, Tokyo, 108-8639, Japan; Human Genome Center, Institute of Medical Science, University of Tokyo, 4-6-1 Shirokanedai, Minato-ku, Tokyo, 108-8639, Japan

**Keywords:** differential gene network analysis, network rewiring, log-likelihood ratio, nonparametric inference, gene prioritization

## Abstract

Understanding phenotype-specific network rewiring is essential for elucidating the molecular mechanisms underlying complex diseases. Although numerous differential network analysis methods have been developed, most focus on only a limited aspect of network information. Consequently, these approaches often fail to jointly characterize regulatory activity and network architecture, provide limited statistical evidence at the network level, and offer little capability for prioritizing key genes that drive phenotype-specific network rewiring. To address these limitations, we developed a novel computational framework, integrated probabilistic differential network analysis (iProDNet), for identifying phenotype-specific regulatory modules under heterogeneous biological conditions. A key feature of iProDNet is the integration of regulatory effects and network topological characteristics into a unified network-aware gene activity score. The framework further transforms gene-level differential activity into network-level statistical evidence using a nonparametric log-likelihood ratio approach and naturally prioritizes genes that contribute to phenotype-specific network rewiring. We evaluated iProDNet through Monte Carlo simulations and compared its performance with existing methods. Across diverse simulation settings, iProDNet consistently achieved superior discriminative performance while maintaining a balanced tradeoff between sensitivity and specificity. We further applied iProDNet to whole-blood RNA-sequencing data generated by the Japan Corona virus disease 2019 (COVID19) Task Force to identify molecular interactions associated with severe COVID19. The proposed framework identified distinct rewired subnetworks related to immune regulation, ribosome-mediated translation, and interferon-driven antiviral responses. Furthermore, iProDNet successfully prioritized biologically relevant markers, including B2M, ISG15, and multiple ribosomal protein genes, all of which have previously been implicated in COVID19 severity and antiviral immunity. These results demonstrate that iProDNet provides a statistically robust and biologically interpretable framework for identifying phenotype-specific regulatory modules and key regulatory drivers. As such, it offers new opportunities for investigating molecular rewiring mechanisms and discovering disease-associated therapeutic targets.

## Introduction

Identifying network rewiring events that underlie phenotypic differences has become an important research topic in systems biology. While conventional transcriptomic analyses primarily focus on differential expression of individual genes, accumulating evidence suggests that disease progression is often accompanied by substantial alterations in network-level regulatory programs. Recent advances in high-throughput transcriptomic technologies have enabled the construction of large-scale gene regulatory networks, enabling the development of computational approaches for identifying phenotype-specific network rewiring events and their key regulatory drivers. A major objective of differential network analysis is to identify gene networks whose architectures differ substantially between phenotypes.

Numerous computational approaches have been proposed for this purpose. DiffGraph identifies network rewiring through differential graphical models by estimating changes in conditional dependency structures between phenotypes [1]. WGCNA constructs weighted co-expression networks and identifies biologically meaningful modules based on pairwise gene correlations [2]. DGCA detects phenotype-specific changes in gene–gene correlation patterns through differential correlation analysis [3]. Recently, nethet was developed to characterize heterogeneous biological network structures in high-dimensional settings [4]. Nonlinear approaches have also been developed for modeling complex biological systems. These include RNDEtree, which combines an ordinary differential equation model with a flexible neural tree [5], and a two-step gene regulatory network inference method integrating time-delayed correlation with a complex-valued S-system [6]. Related computational frameworks have also been applied to complex-valued system identification and post-translational modification site prediction [7, 8]. Recent computational advances have further expanded network-based analyses to single-cell and spatial transcriptomic data. These approaches include graph-based inference of gene regulatory networks [9], integration and interpolation of heterogeneous spatial transcriptomic data [10], and incorporation of intercellular communication into the prediction of intracellular regulatory relationships [11]. Collectively, these developments highlight the importance of integrating heterogeneous regulatory information to characterize phenotype-specific network alterations. Although these methods have demonstrated utility in detecting network differences, existing approaches typically characterize differential networks using only a single aspect of network information, such as topological properties, correlation patterns, or edge-level regulatory changes. Consequently, they often overlook the joint effects of regulatory activity and network architecture, limiting their ability to comprehensively characterize phenotype-specific network rewiring. Furthermore, existing methods generally provide limited statistical evidence at the network level and offer little capability for prioritizing key genes that drive phenotype-specific network rewiring. In addition, many approaches rely on restrictive parametric assumptions that may not adequately capture the complex characteristics of biological network data [12].

To address these challenges, we developed a novel computational framework, integrated probabilistic differential network analysis (iProDNet), for identifying phenotype-specific regulatory modules under heterogeneous biological conditions. A key innovation of iProDNet is the integration of regulatory activity and network topology into a unified network-aware gene activity score. Specifically, regulatory effects are combined with multiple topological characteristics, including hubness, betweenness centrality [13], and PageRank [14], enabling characterization of both functional activity and structural importance within gene regulatory networks. Furthermore, we extend gene-level differential regulatory activity into network-level statistical evidence through a probabilistic framework that aggregates gene-specific signals into a network-level LLR score [12]. Another important feature of iProDNet is its nonparametric likelihood framework. Unlike existing approaches that rely on restrictive distributional assumptions, iProDNet estimates underlying probability densities directly from observed data using kernel density estimation, enabling flexible characterization of complex biological network structures. Furthermore, the proposed framework naturally prioritizes genes according to their contributions to phenotype-specific network rewiring, thereby facilitating the identification of candidate biomarkers and key regulatory drivers. Consequently, iProDNet provides a comprehensive framework for identifying phenotype-specific regulatory modules by prioritizing key regulatory genes and characterizing molecular rewiring associated with complex biological phenotypes. We evaluated the performance of iProDNet through extensive Monte Carlo simulations using cancer-related gene regulatory networks constructed from the cancer cell line encyclopedia (CCLE) dataset. Additionally, we applied iProDNet to whole-blood RNA-sequencing (RNA-seq) data generated by the Japan COVID19 Task Force to identify severe COVID19-specific molecular interplays. We found distinct rewired subnetworks associated with immune regulation, ribosome-mediated translational processes, and interferon-driven antiviral responses. Furthermore, iProDNet successfully prioritized biologically relevant markers, including B2M, ISG15, and several ribosomal protein genes, which have previously been implicated in COVID19 severity and antiviral immunity.

The remainder of this paper is organized as follows: The Materials and methods section introduces the proposed iProDNet framework. The Results section presents Monte Carlo simulation studies and the application of iProDNet to severe COVID19 transcriptomic data. Finally, the Discussion section summarizes the main findings and discusses their implications.

## Materials and methods

The proposed framework consists of the following main steps:

- Estimate phenotype-specific directed gene regulatory networks using $L_{1}$-regularized regression.
- Calculate and integrate regulatory effects and node-centric topological characteristics to obtain network-aware gene activity scores.
- Quantify differential regulatory activity between phenotypes.
- Construct the background distribution through phenotype-label permutation.
- Calculate gene-level LLR scores and aggregate them into a network-level score.
- Assess the statistical significance of the query network, in which its network-level score is compared with the scores of randomly sampled subnetworks of the same size.

Figure 1 provides a schematic overview of these analytical steps.

**Figure 1:**
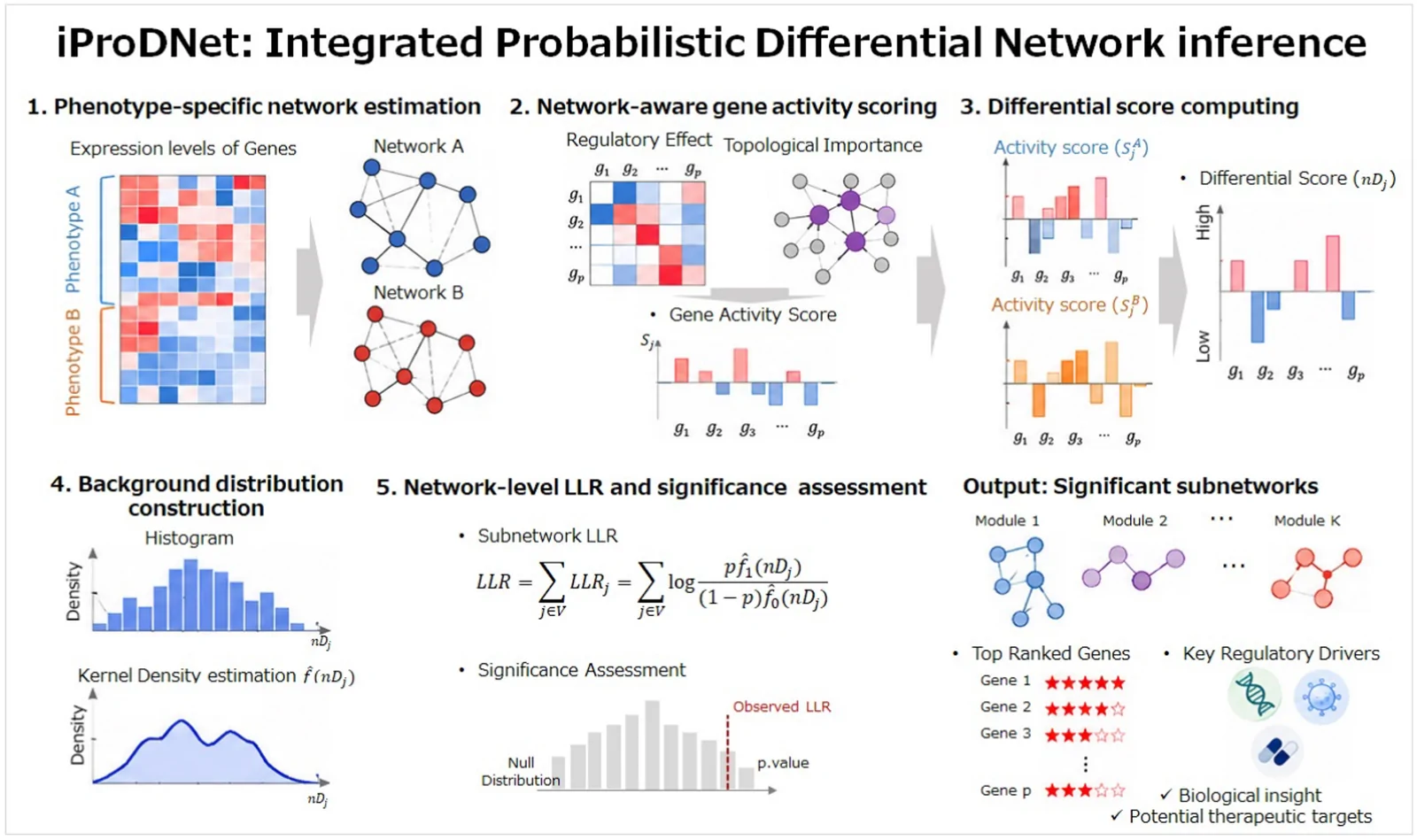
Flowchart of iProDNet for identifying phenotype-specific regulatory modules. The upper panels show phenotype-specific directed network estimation, network-aware gene activity scoring, and differential regulatory activity quantification. The resulting differential activity scores are subsequently used in the lower panels to construct the background distribution through phenotype-label permutation and calculate gene- and network-level log-likelihood ratio (LLR) scores. The statistical significance of the query network is then assessed by comparing its network-level score with those of randomly sampled subnetworks.

### Gene network inference

We consider phenotypes *A* (e.g. cancer) and *B* (e.g. normal). Suppose that $\mathbf{X}^{g}=(X^{g}_{1},\ldots ,X^{g}_{n})^{T}\in \mathbb{R}^{n \times p}$ is an *n × p* data matrix describing the expression levels of *p* genes for *n* cell lines. The directed gene network of the phenotypes *g=A, B* can be represented by the following linear regression model:


(1) \begin{gather*} X_{i\ell}^{g}=\sum_{j \neq \ell}^{p}\beta_{\ell j}^{g}X_{ij}^{g}+\varepsilon_{i\ell}^{g}, \qquad \ell=1,\ldots,p, \quad i=1,\ldots,n \end{gather*}

where $X_{i\ell }^{g}$ is the expression level of the $\ell{\text{ th}}$ gene in the $i{\mathrm{th}}$ cell line, and $\boldsymbol{\varepsilon }^{g}_{\ell }=(\varepsilon _{1\ell }^{g},\ldots ,\varepsilon _{n\ell }^{g})^{T}\in \mathbb{R}^{n}$ is a random error vector corresponding to residual variation. The regression coefficient $\boldsymbol{\beta }_{\ell }^{g}=(\beta _{\ell 1}^{g},\ldots ,\beta _{\ell p}^{g})^{T}$ indicates the edge weights of the $\ell{\mathrm{th}}$ target gene from other *p-1* genes. We consider the following $L_{1}$-type regularization method to estimate the edge weights $\boldsymbol{\beta }^{g}_{\ell }$:


(2) \begin{gather*} \hat{\boldsymbol{\beta}}_{\ell}^{g} = \arg\min_{\boldsymbol{\beta}_{\ell}^{g}} \left\{\frac{1}{2}\sum_{i=1}^{n} \left(X_{i\ell}^{g}-\sum_{j \neq \ell}\beta_{\ell j}^{g}X_{ij}^{g}\right)^{2}+P_{\lambda}\left(\|\boldsymbol{\beta}_{\ell}^{g}\|\right)\right\}, \end{gather*}

where $P_{\lambda }\left (\|\boldsymbol{\beta }_{\ell }^{g}\|\right )$ is the $L_{1}$-type penalty, e.g. lasso[15] or elastic net [16].

### Gene activity scoring via network-centric weighting

To capture both regulatory activity and topological importance, we considered and combined multiple network topological properties into a network-aware gene activity score:

- Regulatory effect [17]
  (3) \begin{gather*} R_{j}^{g}=\sum_{\ell=1}^{p}\left|\beta_{\ell j}^{g}\bar{X}_{j}^{g}\right|,\qquad g=A,B, \end{gather*}
  where $\bar{X}_{j}^{g}$ is the average expression level of the $j{\mathrm{th}}$ gene for phenotype *g=A,B*. A larger value of $R_{j}^{g}$ suggests that the $j{\mathrm{th}}$ gene exerts stronger regulatory influence in phenotype *g*, as it combines increased expression levels with greater topological importance in the gene regulatory network.
- Hubness [17]
  (4) \begin{gather*} H_{j}^{g}=\frac{|V_{j}^{g}|}{|V_{j}^{A}\cup V_{j}^{B}|},\qquad g=A,B, \end{gather*}
  where $0 \le H_{j}^{g} \le 1$, and $V_{j}^{A}$ and $V_{j}^{B}$ are sets of nodes that are directly connected with the $j{\mathrm{th}}$ gene in the networks of phenotypes *A* and *B*, respectively. A large value of $H_{j}^{g}$ indicates that the $j{\mathrm{th}}$ gene maintains a greater number of direct connections relative to the overall network structure, suggesting stronger local connectivity and a more central role within the gene network of phenotype *g*.
- Betweenness centrality [13]
  (5) \begin{gather*} B_{j}^{g}=\frac{1}{(|V^{g}|-1)(|V^{g}|-2)}\sum_{t \neq j \neq u}\frac{a_{tu}^{g}(j)}{a_{tu}^{g}}, \end{gather*}
  where $|V^{g}|$ is a number of nodes in the network of phenotype *g*, $0 \le B_{j}^{g} \le 1$, $a_{tu}^{g}$ is the total number of shortest paths from the $t{\mathrm{th}}$ and $u{\mathrm{th}}$ genes, and $a_{tu}^{g}(j)$ is the number of these paths passing through the $j{\mathrm{th}}$ gene in phenotype *g*. A larger value of $B_{j}^{g}$ indicates that the $j{\mathrm{th}}$ gene occupies a more central position in the communication pathways of the gene network, exerting greater influence on information flow within phenotype *g*.
- PageRank [14]
  To measure the global network centrality representing a gene’s overall regulatory influence, we used the following PageRank measure:
  (6) \begin{gather*} P_{j}^{g}=(1-d)+d\sum_{k \in V_{j}^{g}}\frac{P_{k}^{g}}{L_{k}^{g}}, \end{gather*}
  where $0 \le P_{j}^{g} \le 1$, $L_{k}^{g}$ denotes the number of edges connected to the $k{\mathrm{th}}$ gene in the network of phenotype *g*, and *d* is the damping factor parameter. A high value of $P_{j}^{g}$ indicates that the $j{\mathrm{th}}$ gene occupies a globally influential position within the gene regulatory network, reflecting its importance as a target of regulatory interactions from other influential genes.

#### Network-aware gene activity scoring

Then, we define a network-aware gene activity score by integrating the node-centric characteristics. To ensure comparability across node-centric measures with different scales, we first standardize hubness, betweenness centrality, and PageRank using z-score normalization as follows:


\begin{gather*} Z_{H_{j}}^{g}=\frac{H_{j}^{g}-\mu_{H}^{g}}{\sigma_{H}^{g}}, \quad Z_{B_{j}}^{g}=\frac{B_{j}^{g}-\mu_{B}^{g}}{\sigma_{B}^{g}}, \quad Z_{P_{j}}^{g}=\frac{P_{j}^{g}-\mu_{P}^{g}}{\sigma_{P}^{g}}. \end{gather*}

These quantities are aggregated into a unified weight $W_{j}^{g}$ using the following sigmoid transformation:


(7) \begin{gather*} W_{j}^{g}=\frac{1}{1+\exp\left[-\left(Z_{H_{j}}^{g}+Z_{B_{j}}^{g}+Z_{P_{j}}^{g}\right)\right]}. \end{gather*}

Then we propose the network-aware gene activity score $S_{j}^{g}$ by weighting the regulatory effect with $W_{j}^{g}$,


(8) \begin{gather*} S_{j}^{g}=W_{j}^{g}R_{j}^{g}, \end{gather*}

which integrates both expression-level activity and the topological importance of genes within the regulatory network.

To quantify differential regulatory activity between phenotypes, we define the differential activity score of the $j{\mathrm{th}}$ gene as:


(9) \begin{gather*} D_{j}=S_{j}^{A}-S_{j}^{B}. \end{gather*}

The differential regulation score is normalized to account for differences in location and variability between phenotypes, and to ensure comparability across genes,


(10) \begin{gather*} nD_{j}=\frac{(S_{j}^{A}-S_{j}^{B})-(\mu_{A}-\mu_{B})}{\sqrt{\sigma_{A}^{2}+\sigma_{B}^{2}}}, \end{gather*}

where $\mu _{A}$ ($\mu _{B}$) and $\sigma _{A}^{2}$ ($\sigma _{B}^{2}$) denote the mean and variance of the gene scores under phenotype *A* (*B*), respectively. This normalization accounts for phenotype-specific differences in location and dispersion, enabling a fair comparison of differential regulation signals across genes. Because iProDNet aims to identify networks exhibiting differential regulatory activity between phenotypes, rather than networks preferentially activated in either phenotype, we define the differential gene activity score as the absolute value of the normalized differential regulation score, ($nD_{j}$). Here, the sign of ($nD_{j}$) indicates which phenotype exhibits greater gene activity and does not represent the directionality of regulatory interactions within the network. Hereafter, $nD_{j}$ denotes $|nD_{j}|$ unless otherwise specified.

### Dysfunctional gene network detection

To identify the phenotype specific gene network, we refer to the framework of [12, 18]. We model $nD_{j}$ using a two-component distribution framework consisting of the background $f_{0}$ and real $f_{1}$ distributions. Let *p* denotes the prior probability that $nD_{j}$ originates from $f_{1}$. Differentially regulated gene network identification aims to test the following hypothesis:

- **Null hypothesis ($H_{0}^{G}$)**: The network *G* consists of genes whose activity scores are sampled from the background distribution $f_{0}$. This implies that the network does not exhibit phenotype-specific behavior.
- **Alternative hypothesis ($H_{1}^{G}$)**: The network *G* consists of genes sampled from the real distribution $f_{1}$, indicating significant phenotype-specific molecular interplay.

To test the hypotheses, we consider the following posterior probability in line with [18]


(11) \begin{gather*} P(H_{\delta}^{G}\mid G), \qquad \delta=0,1, \end{gather*}

which measures the strength of evidence supporting hypothesis $H_{\delta }^{G}$ after observing the activity scores within network *G*. Using Bayes’ theorem, the posterior probabilities that an observed activity score $nD_{j}$ originates from the real distribution $f_{1}$ or background distribution $f_{0}$ can be expressed as follows:


\begin{gather*} P(H_{1}^{G} \mid nD_{j})=\frac{p f_{1}(nD_{j})}{p f_{1}(nD_{j})+(1-p)f_{0}(nD_{j})},\\ P(H_{0}^{G} \mid nD_{j})=\frac{(1-p)f_{0}(nD_{j})}{p f_{1}(nD_{j})+(1-p)f_{0}(nD_{j})}. \end{gather*}

Thus, the LLR score of the $j{\mathrm{th}}$ gene (i.e. $nD_{j}$) can be defined as follows:


(12) \begin{gather*} {\mathrm{LLR}}(nD_{j}) =\log\frac{p f_{1}(nD_{j})}{(1-p)f_{0}(nD_{j})}. \end{gather*}

The large value of LLR$(nD_{j})$ indicates stronger evidence that the observed activity score originates from the real distribution, implying phenotype-specific molecular interaction rather than background variation.

Although previous studies assumed that $f_{0}$ and $f_{1}$ follow normal distributions for simplicity [12], biological network data often exhibit complex distributional characteristics that are inadequately captured by a specific parametric distribution. Therefore, imposing a Gaussian assumption may limit the ability to accurately characterize the underlying data structure. To address this limitation, we propose the use of a novel nonparametric approach for computing the LLR score of gene networks. This approach relaxes the Gaussianity assumption and estimates the probability density functions $f_{1}$ and $f_{0}$ directly from the observed data without imposing prior distributional assumptions.

1. Empirical probability density estimation The probability density at a given activity score $D_{j}$ is initially approximated using an empirical histogram approach [19]. By partitioning the observed scores into discrete bins, we define the following density:
  (13) \begin{gather*} \hat{f}(nD_{j}) \approx \frac{ \text{bin count}}{O \times \text{bin width}} \end{gather*}
  where *O* is the total number of observations, $\text{bin count}$ is the number of scores assigned to the histogram interval containing $nD_{j}$, and $\text{bin width}$ is the width of the corresponding histogram interval. This discrete representation allows us to observe the raw distribution of gene activity scores $nD_{j}$ before applying smoothing filters.
2. Generating the background distribution
  To construct the background distribution $f_{0}$, we used phenotype-label permutation. Under the null hypothesis, phenotype labels are exchangeable because gene-expression profiles and network structures are assumed not to be systematically associated with phenotype membership. In each permutation, the phenotype labels of the cell lines were randomly shuffled while preserving the original numbers of cell lines in the two groups. Directed gene regulatory networks were then separately re-estimated for the two permuted groups using the same $L_{1}$-regularized regression procedure (i.e. lasso [15]). For each permutation, the network-aware gene activity scores were recalculated, and the absolute normalized differential activity score $nD_{j}^{*}$ was obtained from the two permuted networks. This procedure was repeated *B* times, and the resulting permuted scores were pooled to estimate the empirical background distribution $f_{0}$. Therefore, $f_{0}$ represents the distribution of differential activity scores expected in the absence of phenotype-specific network regulation, rather than a network without gene–gene interactions.
3. Smoothing via kernel density estimation To address the discontinuity and bin sensitivity of histograms, we apply kernel density estimation to obtain smooth and continuous density functions for both $f_{0}$ and $f_{1}$. The smoothed density function is computed as:
  \begin{gather*} \hat{f}_{\delta}(nD_{j})=\frac{1}{O_{\delta} h_{\delta}}\sum_{m=1}^{O_{\delta}}K\left(\frac{nD_{j}-nD^{\delta}_{m}}{h_{\delta}}\right),\qquad \delta=0,1. \end{gather*}
  where $nD^{\delta }_{m}$ denotes the $m{\mathrm{th}}$ score used to estimate density function $f_{\delta }$, $O_{\delta }$ is the total number of scores used for estimating $f_{\delta }$, *K(· )* denotes the kernel function, and $h_{\delta }$ is the corresponding bandwidth parameter. We employed a Gaussian kernel and optimized the bandwidth parameter $h_{\delta }$ using unbiased cross-validation [20].
4. LLR calculation Using the kernel-smoothed empirical densities $\hat{f}_{0}$ and $\hat{f}_{1}$, we derive the LLR for the $j{\mathrm{th}}$ gene as follows:
  (14) \begin{gather*} {\mathrm{LLR}}(nD_{j}) = \log\left(\frac{p\hat{f}_{1}(nD_{j})}{(1-p)\hat{f}_{0}(nD_{j})}\right), \end{gather*}
  where the prior probability *p* that a gene exhibits phenotype-specific regulatory activity is defined as follows:
  (15) \begin{gather*} p=P(nD_{j}\ge \mu_{0}), \end{gather*}
  where $\mu _{0}$ is mean of $nD_{j}^{*}$, characterizing the expected score distribution under the null hypothesis.

#### Identifying differentially regulated gene networks

The joint probability of the network can be rewritten as the product of individual densities as follows:


(16) \begin{gather*} P(H_{\delta}^{G}\mid G)=\prod_{j \in V}p^{\,i}(1-p)^{1-i}\hat{f}_{\delta}(nD_{j}), \quad \delta=0,1. \end{gather*}

The evidence supporting the alternative hypothesis over the null hypothesis can be quantified by the ratio of posterior probabilities.

We develop the network-level LLR as follows:


(17) \begin{eqnarray*} {\mathrm{nwLLR}}=\log\left[\frac{P(H_{1}^{G}\mid G)}{P(H_{0}^{G}\mid G)}\right]&=\sum_{j \in V}\log\left[\frac{p\hat{f}_{1}(nD_{j})}{(1-p)\hat{f}_{0}(nD_{j})}\right]\\ \nonumber &=\sum_{j \in V}{\mathrm{LLR}}(nD_{j}).\end{eqnarray*}

Based on the derived nwLLR, we define the following decision rule: We accept the alternative hypothesis $H_{1}^{G}$ if and only if the posterior probability of $H_{1}^{G}$ is greater than that of $H_{0}^{G}$,


(18) \begin{gather*} P(H_{1}^{G}\mid G)> P(H_{0}^{G}\mid G) \iff \sum_{j \in V}LLR(nD_{j}) > 0. \end{gather*}

Consequently, gene networks with positive cumulative LLR scores are classified as differentially regulated modules, suggesting the presence of phenotype-specific regulatory rewiring.

## Results

### Network-level differential regulation detection

We evaluated the performance of the proposed iProDNet through extensive Monte Carlo simulations. Gene expression data were obtained from the CCLE dataset available through the DepMap portal (https://depmap.org/portal/), comprising mRNA expression profiles of 19 221 genes across 1406 cancer cell lines. We consider cancer and normal cell lines as two phenotypes and infer phenotype-specific gene networks corresponding to cancer and normal conditions. Network nodes comprise genes involved in cancer-related pathways obtained from the Kyoto Encyclopedia of Genes and Genomes (KEGG) database (https://www.genome.jp/kegg/pathway.html). Table 1 summarizes the cancer-related pathways and associated cell line cohorts used for phenotype-specific gene network inference.

**Table 1:** Composition of pathway gene sets and cell line cohorts

Cancer category	Entry	No. cells	No. genes
Colorectal cancer	hsa05210	85	87
Gastric cancer	hsa05226	48	150
Nonsmall cell lung cancer	hsa05223	156	73
Pancreatic cancer	hsa05212	60	77
Small cell lung cancer	hsa05222	77	93

Expression profiles of *p* genes were used to infer the colorectal cancer gene network from 85 colorectal cancer cell lines. In the simulation study, the total network size was defined as *p* to evaluate performance across different network dimensions. Accordingly, the colorectal cancer network was constructed using all 87 colorectal cancer pathway genes together with *p-87* genes randomly selected from the remaining expression dataset. Although the analysis could be restricted to the 87 pathway genes, the inclusion of additional genes enabled evaluation under larger and more diverse network-size scenarios. For comparison, a normal gene network was inferred using expression profiles from an identical gene set measured in 103 noncancerous cell lines. Then, we applied the proposed iProDNet to identify differentially regulated gene networks. If the colorectal cancer pathway-related network was identified as a colorectal cancer-specific gene network, the result was considered a true positive for differentially regulated gene network identification. To evaluate the spurious detection rate of differentially regulated gene network identification, we constructed two gene networks using expression profiles of randomly selected *p* genes and two randomly selected sets of 85 cell lines. Since both networks were generated under the null setting without phenotype-specific regulatory signals, the identification of a differentially regulated gene network was considered a false positive. The simulation procedure was repeated 50 times. The same simulation framework was applied to gastric, nonsmall cell lung, small cell lung, and pancreatic cancers. For each simulation replicate, *B=20* phenotype-label permutations were performed while preserving the original group sizes. The phenotype-specific directed networks were re-estimated for every permutation using the same $L_{1}$-regularized regression procedure (i.e. lasso [15]). The observed absolute differential activity scores (i.e. $|nD_{j}|$) were used to estimate $f_{1}$, whereas all permuted scores (i.e. $|nD_{j}^{*}|$) were pooled to estimate $f_{0}$. Both densities were estimated using a Gaussian kernel, with the bandwidth selected separately for each distribution by unbiased cross-validation [20]. The prior probability in (15) was estimated as the proportion of genes whose absolute differential activity scores met or exceeded the pooled permutation mean. The network-level score was calculated by summing the gene-level LLR scores, and a network was classified as differentially regulated when its cumulative LLR score was greater than zero. No additional edge-weight or subnetwork-size filtering was applied in the simulation study. We evaluate our strategy against existing differential network methods, including DiffGraph [1], WGCNA [2], DGCA [3], nethet [4], and chNet [21], together with three high-dimensional covariance tests: Li–Chen [22], PE-Cov [23], and Schott [24].

Figure 2 shows the receiver operating characteristic (ROC) curves of each method. For iProDNet, the network-level LLR scores were used as decision statistics, and different thresholds on these scores were varied to obtain the ROC curve. For DiffGraph, WGCNA, DGCA, nethet, Li–Chen, PE-Cov, Schott, and chNet, the decision statistics were defined as the negative logarithm of their corresponding *P*-values. As shown in Fig. 2, iProDNet demonstrated strong and competitive performance across cancer types and network dimensions. Li–Chen and Schott also showed consistently high discrimination, whereas chNet performed well in most settings. PE-Cov exhibited strong performance in several cancer types but showed reduced discrimination in NSCL. The performance of WGCNA, DGCA, and nethet varied across scenarios, with pronounced reductions in the larger gastric cancer networks. DiffGraph generally showed weaker discrimination, with ROC curves frequently approaching the diagonal reference line. Overall, these results support the competitive performance of iProDNet across the evaluated settings, although no single method uniformly outperformed all others. The corresponding area under the ROC curve (AUC) values are summarized in Supplementary Fig. S1, providing a quantitative comparison across cancer types and network dimensions.

**Figure 2:**
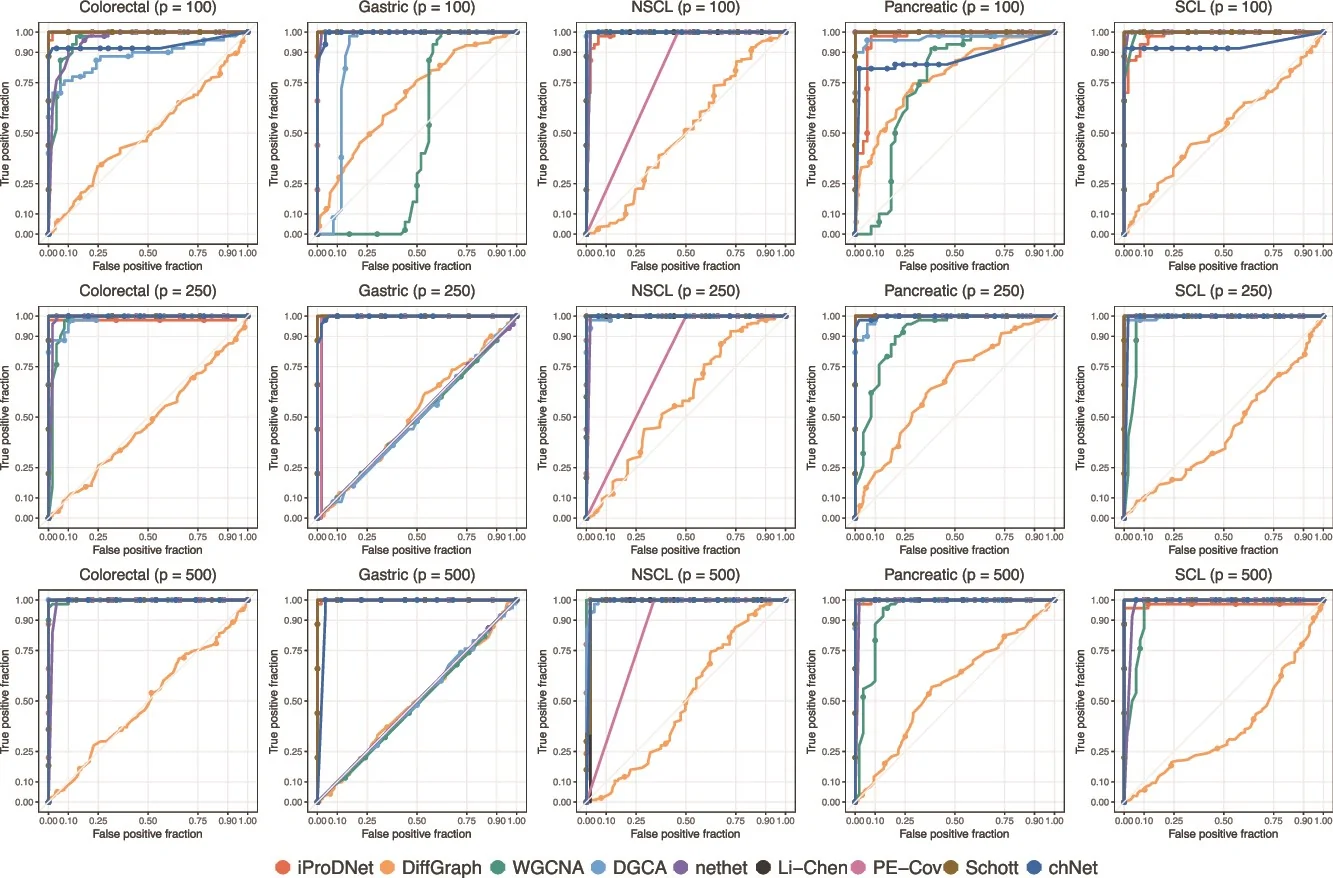
ROC curves comparing iProDNet with DiffGraph, WGCNA, DGCA, nethet, Li–Chen, PE-Cov, Schott, and chNet for differential network detection. Columns represent five cancer types, and rows correspond to network sizes of *p=100*, *250*, and *500*. The horizontal and vertical axes indicate the false positive rate and true positive rate, respectively. The diagonal line represents random discrimination.

We further assessed the performance of the competing methods using several evaluation metrics, including accuracy (Acc), precision (Prec), recall (Recall), F1 score (F1), true positive rate (TPR), and true negative rate (TNR). Table 2 lists the evaluation results based on the metrics.

**Table 2:** Summary of simulation performance across different cancer types and network dimensions (*p=100,250,500*) based on accuracy, precision, recall, F1 score, TPR, and TNR, where ‘–’ denotes conditions under which precision could not be assessed because no positive gene network was identified.

	Methods	No. genes: 100						No. genes: 250						No. genes: 500					
		Acc	Prec	Recall	F1	TPR	TNR	Acc	Prec	Recall	F1	TPR	TNR	Acc	Prec	Recall	F1	TPR	TNR
Colorectal	iProDNet	0.95	0.91	1.00	0.95	1.00	0.90	0.99	1.00	0.98	0.99	0.98	1.00	**1.00**	1.00	1.00	1.00	1.00	1.00
	DiffGraph	0.86	1.00	0.72	0.84	0.72	1.00	0.88	1.00	0.76	0.86	0.76	1.00	0.72	1.00	0.44	0.61	0.44	1.00
	DGCA	0.57	1.00	0.14	0.25	0.14	1.00	0.81	1.00	0.62	0.77	0.62	1.00	0.94	1.00	0.88	0.94	0.88	1.00
	nethet	0.80	0.97	0.62	0.76	0.62	0.98	0.86	0.97	0.74	0.84	0.74	0.98	0.91	0.98	0.84	0.90	0.84	0.98
	WGCNA	0.67	0.90	0.38	0.54	0.38	0.96	0.66	0.94	0.34	0.50	0.34	0.98	0.66	0.94	0.34	0.50	0.34	0.98
	Li-Chen	**0.98**	0.96	1.00	0.98	1.00	0.96	0.98	0.96	1.00	0.98	1.00	0.96	0.97	0.94	1.00	0.97	1.00	0.94
	PE-Cov	**0.98**	0.96	1.00	0.98	1.00	0.96	0.98	0.96	1.00	0.98	1.00	0.96	0.97	0.94	1.00	0.97	1.00	0.94
	Schott	0.97	0.94	1.00	0.97	1.00	0.94	0.97	0.94	1.00	0.97	1.00	0.94	0.96	0.93	1.00	0.96	1.00	0.92
	chNet	0.94	1.00	0.88	0.94	0.88	1.00	**1.00**	1.00	1.00	1.00	1.00	1.00	**1.00**	1.00	1.00	1.00	1.00	1.00
Gastric	iProDNet	**1.00**	1.00	1.00	1.00	1.00	1.00	**0.98**	1.00	0.96	0.98	0.96	1.00	**0.97**	1.00	0.94	0.97	0.94	1.00
	DiffGraph	0.54	1.00	0.08	0.15	0.08	1.00	0.45	0.00	0.00	–	0.00	0.90	0.49	0.00	0.00	–	0.00	0.98
	DGCA	0.50	–	0.00	–	0.00	1.00	0.50	–	0.00	–	0.00	1.00	0.44	0.33	0.12	0.18	0.12	0.76
	nethet	0.68	1.00	0.36	0.53	0.36	1.00	0.50	0.50	0.76	0.60	0.76	0.24	0.50	0.50	0.86	0.63	0.86	0.14
	WGCNA	0.50	–	0.00	–	0.00	1.00	0.50	–	0.00	–	0.00	1.00	0.50	–	0.00	–	0.00	1.00
	Li-Chen	0.98	0.96	1.00	0.98	1.00	0.96	**0.98**	0.96	1.00	0.98	1.00	0.96	0.95	0.91	1.00	0.95	1.00	0.90
	PE-Cov	0.98	0.96	1.00	0.98	1.00	0.96	**0.98**	0.96	1.00	0.98	1.00	0.96	0.95	0.91	1.00	0.95	1.00	0.90
	Schott	0.96	0.93	1.00	0.96	1.00	0.92	**0.98**	0.96	1.00	0.98	1.00	0.96	0.95	0.91	1.00	0.95	1.00	0.90
	chNet	0.85	0.97	0.72	0.83	0.72	0.98	0.95	1.00	0.90	0.95	0.90	1.00	0.95	0.96	0.94	0.95	0.94	0.96
NSCL	iProDNet	0.95	0.98	0.92	0.95	0.92	0.98	0.99	1.00	0.98	0.99	0.98	1.00	0.97	1.00	0.94	0.97	0.94	1.00
	DiffGraph	0.95	1.00	0.90	0.95	0.90	1.00	0.90	1.00	0.80	0.89	0.80	1.00	0.77	1.00	0.54	0.70	0.54	1.00
	DGCA	0.91	1.00	0.82	0.90	0.82	1.00	0.84	1.00	0.68	0.81	0.68	1.00	0.90	1.00	0.80	0.89	0.80	1.00
	nethet	0.99	0.98	1.00	0.99	1.00	0.98	0.99	0.98	1.00	0.99	1.00	0.98	0.99	0.98	1.00	0.99	1.00	0.98
	WGCNA	0.99	0.98	1.00	0.99	1.00	0.98	**1.00**	1.00	1.00	1.00	1.00	1.00	**1.00**	1.00	1.00	1.00	1.00	1.00
	Li-Chen	0.95	0.91	1.00	0.95	1.00	0.90	0.99	0.98	1.00	0.99	1.00	0.98	0.96	0.93	1.00	0.96	1.00	0.92
	PE-Cov	0.74	0.66	1.00	0.79	1.00	0.48	0.75	0.67	1.00	0.80	1.00	0.50	0.82	0.74	1.00	0.85	1.00	0.64
	Schott	0.94	0.89	1.00	0.94	1.00	0.88	0.97	0.94	1.00	0.97	1.00	0.94	0.94	0.89	1.00	0.94	1.00	0.88
	chNet	**1.00**	1.00	1.00	1.00	1.00	1.00	0.99	1.00	0.98	0.99	0.98	1.00	0.99	0.98	1.00	0.99	1.00	0.98
Pancreatic	iProDNet	0.91	0.94	0.88	0.91	0.88	0.94	**1.00**	1.00	1.00	1.00	1.00	1.00	0.95	1.00	0.90	0.95	0.90	1.00
	DiffGraph	0.85	1.00	0.70	0.82	0.70	1.00	0.66	1.00	0.32	0.48	0.32	1.00	0.85	1.00	0.70	0.82	0.70	1.00
	DGCA	0.74	1.00	0.48	0.65	0.48	1.00	0.84	1.00	0.68	0.81	0.68	1.00	0.87	1.00	0.74	0.85	0.74	1.00
	nethet	0.84	1.00	0.68	0.81	0.68	1.00	**1.00**	1.00	1.00	1.00	1.00	1.00	0.99	0.98	1.00	0.99	1.00	0.98
	WGCNA	0.49	0.00	0.00	–	0.00	0.98	0.51	1.00	0.02	0.04	0.02	1.00	0.51	1.00	0.02	0.04	0.02	1.00
	Li-Chen	**0.98**	0.96	1.00	0.98	1.00	0.96	0.98	0.96	1.00	0.98	1.00	0.96	0.98	0.96	1.00	0.98	1.00	0.96
	PE-Cov	**0.98**	0.96	1.00	0.98	1.00	0.96	0.98	0.96	1.00	0.98	1.00	0.96	0.98	0.96	1.00	0.98	1.00	0.96
	Schott	0.96	0.93	1.00	0.96	1.00	0.92	0.98	0.96	1.00	0.98	1.00	0.96	0.96	0.93	1.00	0.96	1.00	0.92
	chNet	0.85	0.97	0.72	0.83	0.72	0.98	0.95	1.00	0.90	0.95	0.90	1.00	**1.00**	1.00	1.00	1.00	1.00	1.00
SCL	iProDNet	0.92	0.98	0.86	0.91	0.86	0.98	**1.00**	1.00	1.00	1.00	1.00	1.00	0.96	1.00	0.92	0.96	0.92	1.00
	DiffGraph	**1.00**	1.00	1.00	1.00	1.00	1.00	**1.00**	1.00	1.00	1.00	1.00	1.00	**1.00**	1.00	1.00	1.00	1.00	1.00
	DGCA	0.99	1.00	0.98	0.99	0.98	1.00	0.98	1.00	0.96	0.98	0.96	1.00	**1.00**	1.00	1.00	1.00	1.00	1.00
	nethet	0.93	1.00	0.86	0.92	0.86	1.00	0.96	1.00	0.92	0.96	0.92	1.00	0.94	0.96	0.92	0.94	0.92	0.96
	WGCNA	0.86	0.95	0.76	0.84	0.76	0.96	0.68	0.91	0.40	0.56	0.40	0.96	0.68	0.91	0.40	0.56	0.40	0.96
	Li-Chen	0.97	0.94	1.00	0.97	1.00	0.94	0.97	0.94	1.00	0.97	1.00	0.94	0.98	0.96	1.00	0.98	1.00	0.96
	PE-Cov	0.97	0.94	1.00	0.97	1.00	0.94	0.97	0.94	1.00	0.97	1.00	0.94	0.98	0.96	1.00	0.98	1.00	0.96
	Schott	0.96	0.93	1.00	0.96	1.00	0.92	0.96	0.93	1.00	0.96	1.00	0.92	0.98	0.96	1.00	0.98	1.00	0.96
	chNet	0.96	1.00	0.92	0.96	0.92	1.00	0.99	0.98	1.00	0.99	1.00	0.98	**1.00**	1.00	1.00	1.00	1.00	1.00
Average	iProDNet	**0.95**	0.96	0.93	0.94	0.93	0.96	**0.99**	1.00	0.98	0.99	0.98	1.00	0.97	1.00	0.94	0.97	0.94	1.00
	DiffGraph	0.84	1.00	0.68	0.75	0.68	1.00	0.78	0.80	0.58	0.81	0.58	0.98	0.77	0.80	0.54	0.78	0.54	1.00
	DGCA	0.74	1.00	0.48	0.70	0.48	1.00	0.79	1.00	0.59	0.84	0.59	1.00	0.83	0.87	0.71	0.77	0.71	0.95
	nethet	0.85	0.99	0.70	0.80	0.70	0.99	0.86	0.89	0.88	0.88	0.88	0.84	0.87	0.88	0.92	0.89	0.92	0.81
	WGCNA	0.70	0.71	0.43	0.79	0.43	0.98	0.67	0.96	0.35	0.52	0.35	0.99	0.67	0.96	0.35	0.52	0.35	0.99
	Li-Chen	0.97	0.95	1.00	0.97	1.00	0.94	0.98	0.96	1.00	0.98	1.00	0.96	0.97	0.94	1.00	0.97	1.00	0.94
	PE-Cov	0.93	0.90	1.00	0.94	1.00	0.86	0.93	0.90	1.00	0.94	1.00	0.86	0.94	0.90	1.00	0.95	1.00	0.88
	Schott	0.96	0.92	1.00	0.96	1.00	0.92	0.97	0.95	1.00	0.97	1.00	0.94	0.96	0.92	1.00	0.96	1.00	0.92
	chNet	0.92	0.99	0.85	0.91	0.85	0.99	0.98	1.00	0.96	0.97	0.96	1.00	**0.99**	0.99	0.99	0.99	0.99	0.99

Bold values indicate the best performance within each cancer type and network size.

Consistent with the results of ROC and AUC analyses, iProDNet generally achieved superior performance across different cancer types and network dimensions. Notably, iProDNet maintained balanced true positive and true negative rates, resulting in consistently high accuracy and F1 scores across simulation settings. Li–Chen, PE-Cov, and Schott generally achieved high sensitivity but variable specificity, with PE-Cov showing increased false positives in NSCL. chNet performed well overall, although recall was lower in some smaller networks. DiffGraph, DGCA, and WGCNA tended to exhibit high specificity but low sensitivity, whereas nethet showed greater variability across scenarios. In gastric cancer simulations, iProDNet maintained strong performance across network dimensions, while chNet improved with increasing network size and nethet showed reduced specificity in larger networks Overall, these results demonstrate the competitive and balanced performance of iProDNet across the evaluated settings, while highlighting scenario-dependent strengths among the comparative methods.

In order to further evaluate the individual contribution of each topological feature, we performed ablation experiments by separately removing hubness, betweenness centrality, and PageRank from iProDNet. All other components, simulation datasets, parameter settings, and evaluation procedures were kept identical to those used for the complete model. The contribution of each feature was quantified as the percentage reduction in performance relative to the complete iProDNet model:


(19) \begin{gather*} \mathrm{Reduction}\, (\%) = \frac{M_{\mathrm{full}}-M_{\mathrm{ablated}}} {M_{\mathrm{full}}} \times 100, \end{gather*}

where $M_{\mathrm{full}}$ and $M_{\mathrm{ablated}}$ denote the performance of the complete and ablated models, respectively. The percentage reductions were calculated separately for each cancer-specific simulation setting and then averaged across the five cancer types.

As shown in Table 3, the ablation results revealed distinct contributions of the three topological features across different network dimensions. Hubness contributed primarily to performance in smaller networks, whereas the contribution of PageRank increased markedly with network dimensionality. Betweenness centrality showed a relatively smaller overall contribution but provided complementary benefits in higher-dimensional settings. Collectively, these results demonstrate that the three topological features capture complementary aspects of network organization, with their relative contributions varying according to network dimensionality.

**Table 3:** Percentage reduction in accuracy and F1 score after removing individual topological features from iProDNet. Positive values indicate performance deterioration, whereas negative values indicate a slight performance improvement

Removed Features	No. genes: 100		No. genes: 250		No. genes: 500	
	Acc	F1	Acc	F1	Acc	F1
Hubness	8.67	13.45	2.22	2.53	3.92	4.96
Betweenness	0.85	1.42	−0.20	−0.20	3.51	4.54
PageRank	2.54	3.39	9.48	11.78	17.53	26.37

### Gene- and edge-level evaluation with known ground truth

To evaluate gene- and edge-level discrimination, we generated synthetic expression data from two phenotype-specific networks with known ground truth. A random directed network with *p=100, 250, 500* genes and an edge density of *1%* was constructed for phenotype *A*. Nonzero coefficients were assigned random signs with equal probability and magnitudes sampled uniformly from *0.30* to *0.80*. We selected *25%* of all genes as differential regulators. The network for phenotype *B* was constructed by increasing the magnitudes of selected regulators’ edge weights or deleting these edges. Expression data were generated as


\begin{gather*} \mathbf{X}^{g}= \mathbf{E}^{g}(\mathbf{I}_{p}-\mathbf{B}^{g})^{-1} + \mathbf{1}_{n_{g}}(\boldsymbol{\delta}^{g})^{\top}, \quad g\in\{A,B\}, \end{gather*}

where $E^{g}_{ij}\overset{\mathrm{iid}}{\sim }\mathcal{N}(5,1)$ and $\mathbf{B}^{g}$ is the directed coefficient matrix with entry $B^{g}_{jk}$ representing the effect of gene *j* on gene *k*. Each phenotype contained $n_{g}=100$ samples. The selected regulators and the modified or deleted edges defined the gene- and edge-level positive labels, respectively. Networks were re-estimated from the generated expression data, and AUPR was calculated separately at the gene and edge levels for each of 50 simulation replicates.

For gene- and edge-level AUPR evaluation, we compared iProDNet with WGCNA, DGCA, chNet, and DiffGraph. For iProDNet, genes were ranked using gene-level LLR scores, while edges were ranked using absolute differences in estimated regulatory effects in (3). The comparative methods were evaluated using their edge-level scores and gene-level scores obtained directly or derived from network summaries. Li–Chen, PE-Cov, Schott, and nethet were excluded from this evaluation because the implementations used in our benchmark provided only network-level statistics or *P*-values, rather than the individual gene- or edge-level scores required for AUPR calculation. Table 4 shows the AUPR values.

**Table 4:** Gene- and edge-level area under the precision–recall curve (AUPR) values for iProDNet and four comparative methods across three network dimensions (*p=100,250,500*). The highest AUPR in each row is shown in bold

Level	No. genes	iProDNet	WGCNA	DGCA	chNet	DiffGraph
Edges	100	0.025	0.178	**0.238**	0.167	0.017
	250	**0.413**	0.119	0.104	0.036	0.008
	500	**0.359**	0.090	0.045	0.015	0.007
Genes	100	**0.391**	0.325	0.344	0.364	0.249
	250	**0.394**	0.336	0.330	0.327	0.229
	500	0.391	0.331	0.391	**0.438**	0.258

As shown in Table 4, iProDNet achieved the highest gene-level AUPR in the smaller and intermediate networks, whereas chNet performed best in the largest networks. For edge-level evaluation, DGCA achieved the highest AUPR in the smallest networks, while iProDNet yielded the highest values in the two larger network settings. Overall, these results support the utility of iProDNet for prioritizing differential genes, with promising auxiliary edge-level performance in larger networks.

### Network rewiring associated with severe COVID19

We applied the proposed framework to whole-blood RNA-seq data generated by the Japan COVID19 Task Force [25] to identify molecular networks characterizing severe COVID19. The dataset consisted of 1102 genotyped samples classified as asymptomatic (*n=71*), mild (*n=241*), severe (*n=404*), and critical (*n=303*). To investigate host molecular mechanisms associated with viral infection, we included all 12 pathways classified under the KEGG category “Human Diseases: Infectious disease: viral”:

- Coronavirus disease-COVID19 (ko05171, 219 genes)
- Human T-cell leukemia virus 1 infection (ko05166, 191 genes)
- Human immunodeficiency virus 1 infection (ko05170, 163 genes)
- Hepatitis B (ko05161, 132 genes)
- Hepatitis C (ko05160, 112 genes)
- Influenza A (ko05164, 128 genes)
- Measles (ko05162, 117 genes)
- Herpes simplex virus 1 infection (ko05168, 149 genes)
- Human cytomegalovirus infection (ko05163, 192 genes)
- Kaposi sarcoma-associated herpesvirus infection (ko05167, 169 genes)
- Epstein–Barr virus infection (ko05169, 172 genes)
- Human papillomavirus infection (ko05165, 231 genes)

The union of genes contained in these pathways was used to define a nonredundant candidate node set for network analysis, with genes shared by multiple pathways included only once.

For gene network construction, patients classified as asymptomatic (Level 1) or mild (Level 2) were grouped as nonsevere COVID19 cases, whereas patients classified as critical (Level 4) were grouped as severe COVID19 cases. Directed gene regulatory networks were separately estimated for the severe and nonsevere COVID19 groups using the $L_{1}$-regularization method (i.e. lasso [15]). For each target gene, its expression was regressed on the expression levels of all other genes, with a nonzero regression coefficient defining a directed edge from the predictor gene to the target gene. The regularization parameter was selected using the Bayesian Information Criterion (BIC) [26].

When all inferred interactions were retained, the resulting dense network was dominated by a single large component, providing few distinct molecular modules for downstream analysis. Thus, we retained only the top 10% of edges by absolute weight to limit weak regulatory associations and identify interpretable network communities. Using the Louvain algorithm, the severe COVID19 network was partitioned into communities, of which seven contained >20 genes and were retained as candidate subnetworks for subsequent iProDNet analysis. For each candidate subnetwork, the background distribution $f_{0}$ was constructed using 20 phenotype-label permutations. The absolute normalized differential activity scores obtained from the 20 permutations were pooled to estimate $f_{0}$, whereas the observed absolute differential activity scores were used to estimate $f_{1}$. Both distributions were estimated using Gaussian kernel density estimation, with the bandwidth selected separately for each distribution using unbiased cross-validation (UCV) [20]. The prior probability was estimated as the proportion of genes whose observed absolute differential activity scores met or exceeded the pooled mean of absolute permutation scores.

Table 5 summarizes the network-level LLR scores, empirical *P*-values based on 200 randomly sampled subnetworks of the same size, FDR-adjusted *P*-values, and structural characteristics of the seven subnetworks identified from the severe COVID19-specific gene regulatory network.

**Table 5:** Characteristics and statistical significance of the seven subnetworks identified from the severe COVID19 gene network. Empirical *P*-values were calculated using permutation tests, and FDRs were obtained using the Benjamini–Hochberg correction across the seven subnetworks. SV and Non-SV denote severe and nonsevere COVID19, respectively. Positive network-level LLR scores are shown in bold

Network	LLR	P-value	FDR	No. edges		No. genes	
				SV	Non-SV	SV	Non-SV
1	*-132.6*	0.95	0.95	119	33	75	36
2	**257.7**	*<0.01*	0.03	78	21	59	26
3	*-19.1*	0.93	0.95	17	9	18	10
4	**6.0**	0.12	0.21	116	57	40	36
5	**35.2**	0.01	0.03	157	80	47	39
6	**35.4**	0.01	0.03	44	19	35	19
7	*-17.52*	0.46	0.65	27	7	23	11

As shown in Table 5, the subnetworks generally contained considerably more regulatory interactions and genes in the severe COVID19 network than in the nonsevere network. Therefore, severe COVID19 may be associated with the emergence of additional regulatory interactions and the recruitment of genes within specific molecular modules. Networks 2, 4, 5, and 6 exhibited positive network-level LLR scores; however, after FDR correction, only Networks 2, 5, and 6 remained significant at an FDR threshold of 0.05. In particular, Network 2 showed the largest network-level LLR score, providing the strongest evidence of severe COVID19-specific network rewiring.

To further characterize the biological functions of severe COVID19-specific gene networks, we performed gene ontology (GO) enrichment analysis for Networks 2, 4, 5, and 6, which exhibited significant network rewiring. For each network, the constituent genes were used as the target gene set, whereas all genes involved in the 12 viral infection-related KEGG pathways were used as the background gene set. Figure 3 presents the top five significantly enriched GO terms identified per network. As shown in Fig. 3, the GO enrichment analysis revealed distinct biological functions across the severe COVID19-specific subnetworks. No significantly enriched GO terms were identified for Network 2 with q-value *<0.01*. Conversely, Networks 4 and 5 exhibited highly similar enrichment patterns, with significant enrichment of ribosome- and translation-related GO terms, including *cytoplasmic translation, ribosome, ribosomal subunit*, and *cytosolic ribosome*. Thus, dysregulation of translational and protein synthesis-related processes may represent an important molecular feature of severe COVID19. This is consistent with previous studies demonstrating that SARS-CoV-2 extensively exploits host translational machinery and ribosome-associated pathways to facilitate viral replication and protein synthesis [27, 28]. Network 6 showed a distinct functional profile characterized by significant enrichment of antiviral response-related GO terms, including *response to virus, defense response to virus, defense response to symbiont, negative regulation of viral process*, and *negative regulation of viral genome replication*. These results are consistent with the interferon-associated regulatory module identified in Network 6 and support the involvement of antiviral defense mechanisms in severe COVID19-specific molecular interplays [29].

**Figure 3:**
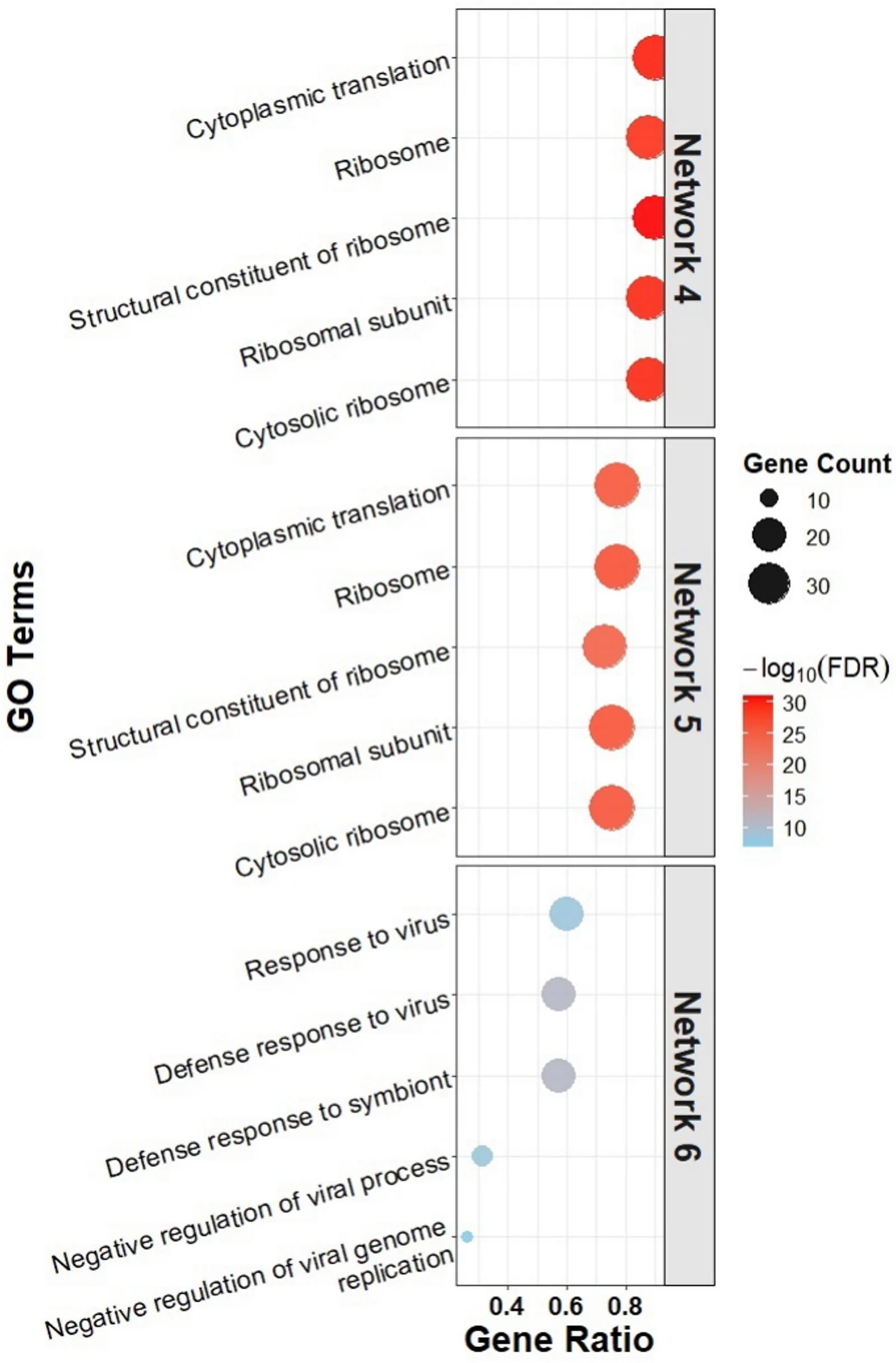
GO enrichment analysis of severe COVID19-specific subnetworks. Significantly enriched GO terms identified from Networks 4, 5, and 6. Bubble size represents the number of genes associated with each GO term. Conversely, color indicates enrichment significance measured by −log10(FDR). Networks 4 and 5 were enriched for ribosome- and translation-related processes, whereas Network 6 was enriched for antiviral response-related processes.

Figure 4 shows the severe COVID19-specific molecular interplays extracted from subnetworks with positive network-level LLR scores. For each selected subnetwork, the top 50 regulatory interactions were retained according to the absolute edge weights and displayed as directed networks. Notably, the strongest regulatory interactions were concentrated around a small number of hub genes within each subnetwork. Specifically, B2M, RPL7, RPL2, and ISG15 were associated with the strongest incoming and/or outgoing regulatory interactions in Networks 2, 4, 5, and 6, respectively. A prominent feature was observed in Networks 4 and 5, which were almost exclusively composed of ribosomal protein genes, including multiple members of the RPL and RPS gene families. In contrast, Network 2 was characterized by hub-like regulatory structures centered on VIM and SPI1. These genes were connected to numerous immune- and inflammation-related genes, including IL18, CD44, CTNNB1, STAT5A, SOCS3, TNFRSF1B, and JAK3, suggesting extensive rewiring of immune regulatory programs in severe COVID19 [30–32]. The biological relevance of Network 2 is further supported by previous studies reporting that VIM contributes to SARS-CoV-2 host–cell interactions and that SPI1 acts as a major transcriptional regulator of COVID19-associated gene expression programs [30, 33]. Network 6 revealed a distinct interferon-associated regulatory module involving ISG15, OASL, IFIH1, IFIT1, EIF2AK2, STAT1, STAT2, and IRF7. Many of these genes are well-established interferon-stimulated genes that participate in antiviral defense responses [34, 35]. Notably, ISG15 and OASL occupied central positions within the network and were connected by strong regulatory interactions, highlighting a potential role of interferon-mediated signaling in the molecular pathogenesis of severe COVID19.

**Figure 4:**
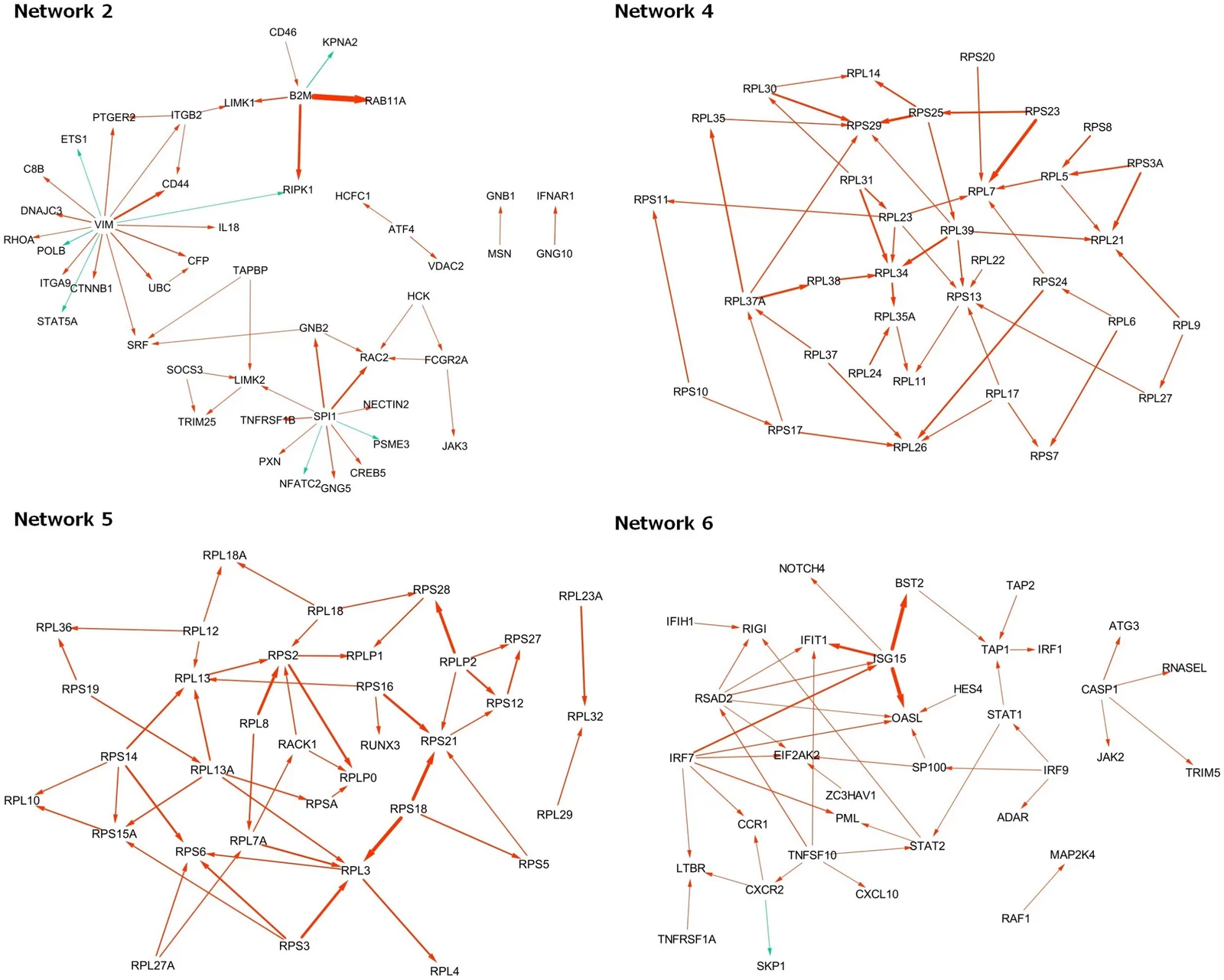
Severe COVID19-specific molecular networks identified from the critical COVID19 gene network. Nodes represent genes, and directed edges represent estimated regulatory interactions. Blue and red edges denote positive and negative regulatory coefficients, respectively, while edge thickness is proportional to the absolute magnitude of the corresponding regulatory coefficient.

Then, we focused on the top five genes with the highest gene-level LLR scores within each severe COVID19-specific subnetwork (Table 6). The identified genes were highly consistent with the structural and functional characteristics of the corresponding subnetworks. Notably, the top-ranked genes were closely associated with the major structural features observed in Fig. 4. In Network 2, B2M, which exhibited the highest gene-level LLR score, was connected to several of the strongest regulatory interactions in the subnetwork, including the prominent B2M–RAB11A and B2M–RIPK1 interactions. Similarly, in Network 6, ISG15, the highest-ranked gene according to the gene-level LLR score, occupied a central position within the interferon-associated regulatory module and was linked to multiple high-weight regulatory interactions involving BST2, OASL, and IFIT1.

- B2M
  Increased serum B2M levels have been associated with COVID19 severity and adverse clinical outcomes [36–38]. Consistent with these observations, B2M exhibited the highest gene-level LLR score in Network 2 and occupied a central position within the severe COVID19-specific regulatory module.
- Ribosome gene family
  Ribosomal protein genes are crucial in SARS-CoV-2 replication and COVID19 pathogenesis, and their dysregulation has been associated with persistent viral infection and immunosuppression [39–41]. These findings support the ribosome- and translation-related subnetworks identified in Networks 4 and 5.
- ISG15
  ISG15 has been associated with SARS-CoV-2-induced hyperinflammation and COVID19 severity [42–44]. Consistent with these findings, ISG15 exhibited the highest gene-level LLR score in Network 6 and occupied a central position within the interferon-associated regulatory module.

**Table 6:** Top five genes ranked by gene-level LLR scores in COVID19 severe-specific subnetworks

Rank	Network 2	Network 4	Network 5	Network 6
1	B2M	RPS25	RPS27	ISG15
2	UBC	RPL37A	RPL19	IRF1
3	GNB2	RPS17	RPS6	BST2
4	RAB11A	RPL39	RPL18A	CXCR2
5	RAC2	RPL35	RPLP2	OASL

These findings demonstrate that the proposed gene-level LLR score successfully prioritizes genes that are strongly involved in severe COVID19-specific regulatory interactions and occupy central positions within the corresponding rewired subnetworks. Considering that several of the identified genes have previously been associated with COVID19 severity, modulation of their activity may represent a promising strategy for mitigating severe disease progression. Consequently, these markers may provide valuable clues for the development of therapeutic interventions targeting severe COVID19-associated molecular dysregulation.

#### Independent validation of COVID19-related subnetworks and key regulatory genes

To validate the robustness of the COVID19-related subnetworks and key regulatory genes identified in the discovery cohort, we further analyzed an independent whole-blood RNA-seq dataset, GSE157103 [45]. This validation cohort comprised 100 patients with COVID19, including 50 patients admitted to the intensive care unit (ICU) and 50 non-ICU patients, who were classified as the severe and nonsevere groups, respectively. The seven subnetworks were identified in the discovery cohort by retaining the top 10% of edges by absolute weight and applying Louvain community detection, with only communities containing >20 genes retained. Their constituent genes were then used as predefined target gene sets for the same network inference and evaluation procedures in the independent cohort. Table 7 summarizes the network characteristics identified in the independent GSE157103 cohort. In the primary analysis, Networks 2, 4, 5, and 6 were identified as representative of severe COVID19. As shown in Table 7, Networks 2 and 6 were replicated in the independent cohort, exhibiting positive LLR scores and consistently greater regulatory connectivity in severe patients. These results support the robustness of their severity-associated network rewiring. We, therefore, further examined the enriched biological functions and top-ranked hub genes of Networks 2 and 6 to assess the reproducibility of their functional characteristics and key regulatory genes.

**Table 7:** Characteristics and statistical significance of the seven COVID19-related subnetworks evaluated in the independent GSE157103 cohort. Empirical *P*-values were obtained using permutation tests, and FDRs were calculated using the Benjamini–Hochberg correction across the seven subnetworks. The significant positive LLR score is shown in bold

Network	LLR	P-value	FDR	No. edges		No. genes	
				SV	Non-SV	SV	Non-SV
1	*-61.3*	1.00	1.00	124	20	72	26
2	**91.7**	*<.01*	0.03	59	7	55	13
3	*-51.5*	.99	1.00	57	7	46	13
4	*-31.3*	.94	1.00	78	11	49	18
5	*-103.9*	1.00	1.00	130	27	82	34
6	**51.7**	.02	0.07	243	58	79	52
7	*-36.1*	.91	1.00	130	41	56	41

For the independently validated Networks 2 and 6, we further performed GO enrichment analysis to assess the reproducibility of their biological functions (Table 8). Consistent with the primary analysis, Network 2 showed no significantly enriched GO terms. In contrast, Network 6 was significantly enriched for cytoplasmic translation, ribosome, ribosomal subunit, cytosolic ribosome, and structural constituent of ribosome in the independent cohort. Notably, all five terms had also been significantly enriched in the severe-COVID19-related subnetworks identified in the primary analysis. This overlap demonstrates the reproducibility of the ribosome- and translation-related functional signature across independent patient cohorts, further supporting the functional robustness of the identified COVID19-related network rewiring.

**Table 8:** Top five GO terms enriched in Network 6 in the independent cohort and their correspondence with the primary analysis

Rank	GO term	Primary analysis
1	Cytoplasmic translation	Networks 4 and 5
2	Ribosome	Networks 4 and 5
3	Ribosomal subunit	Networks 4 and 5
4	Cytosolic ribosome	Networks 4 and 5
5	Structural constituent of ribosome	Networks 4 and 5

We also identified key regulatory genes by ranking genes according to their gene-level LLR scores. As shown in Table 9, the highest-ranked genes included RAC2 in Network 2 and multiple ribosomal protein genes, including RPS17, RPS12, RPS6, RPL27, and RPL12, in Network 6. Notably, the RAC2 and ribosomal protein families were also identified among the key regulatory genes in the primary analysis. This gene-family-level concordance, together with the replicated ribosome- and translation-related GO terms, supports the robustness of the principal regulatory and functional characteristics of these severe-COVID19-related subnetworks across independent patient cohorts.

**Table 9:** Top five genes ranked by gene-level LLR scores in the independently validated Networks 2 and 6

Rank	Network 2	Network 6
1	GNG10	RPS17
2	RAC2	RPS12
3	PXN	RPS6
4	ITGAL	RPL27
5	CFLAR	RPL12

Collectively, these results provide independent support for the reproducibility of Networks 2 and 6. Their positive LLR scores, increased regulatory connectivity in severe patients, overlapping ribosome- and translation-related functions, and recurrence of the RAC and ribosomal protein families indicate partial preservation of their principal regulatory characteristics across cohorts. Although Networks 4 and 5 and the exact rankings of individual genes were not fully replicated, the observed network-, functional-, and gene-family-level concordance supports the robustness of selected severe-COVID19-related regulatory patterns while also reflecting cohort-specific heterogeneity.

## Discussion

We developed a novel computational framework, iProDNet, for identifying phenotype-specific regulatory modules under heterogeneous biological conditions. Unlike existing differential network approaches that primarily focus on changes in differential correlations or edge-level rewiring, iProDNet integrates various regulatory activity and network topologies into a unified network-aware gene activity score. Specifically, regulatory effects were combined with multiple topological characteristics allowing the proposed framework to capture both functional activity and structural importance of genes within gene regulatory networks. This network-aware representation enables a more comprehensive characterization of molecular rewiring associated with disease phenotypes.

Extensive Monte Carlo simulations demonstrated the practical utility of the proposed framework. Across diverse cancer-related pathways and network dimensions, iProDNet consistently outperformed existing approaches. Application of iProDNet to severe COVID19 transcriptomic data identified distinct rewired subnetworks enriched for translational regulation and interferon-mediated antiviral responses. The proposed framework additionally prioritized key genes, including B2M, ISG15, OASL, IRF1, and multiple ribosomal proteins, which have previously been implicated in COVID19 severity and antiviral immunity. These findings demonstrate the capacity of iProDNet t identify disease-associated regulatory modules and biologically meaningful candidate markers from complex gene regulatory networks. Overall, iProDNet provides a statistically robust and biologically interpretable framework for identifying phenotype-specific regulatory modules and prioritizing key regulatory genes.

We anticipate that iProDNet will be a valuable tool for uncovering disease-associated regulatory mechanisms and guiding the discovery of therapeutic targets across various complex diseases.

The thresholds used to retain the top 10% of edges and communities containing >20 genes may affect the resulting network structure and the number and composition of the subnetworks identified. Accordingly, the findings reported in this study should be interpreted in the context of these prespecified criteria. Establishing objective and data-driven criteria for network sparsification and subnetwork selection remains an important direction for future research.

**Key Points**

- Novel computational framework for identifying differentially regulated gene networks, integrated probabilistic differential network analysis (iProDNet), is introduced.
- iProDNet integrates regulatory activity and network topology to identify phenotype-specific regulatory modules and key regulatory genes.
- The proposed framework successfully detects severe COVID19-specific subnetworks associated with immune regulation, translational processes, and antiviral responses.
- iProDNet is a powerful tool for investigating molecular network rewiring and discovering disease-associated regulatory mechanisms.

## Supplementary Material

Supplementary_Figure_S1_bbag565

## Data Availability

The toy dataset and tutorial scripts are publicly available at: https://github.com/HeewonGitHub/iProDNet/tree/IProDNet_v1.
