# Supplementary material for: iProDNet: integrated probabilistic differential network inference under heterogeneous biological conditions
Source: Brief Bioinform. 2026 Oct 8;27(5):bbag565. doi: 10.1093/bib/bbag565 (PMC13647284; doi:10.1093/bib/bbag565)
Supplement: Supplementary file 1 [file supplementary_figure_s1_bbag565.pdf]

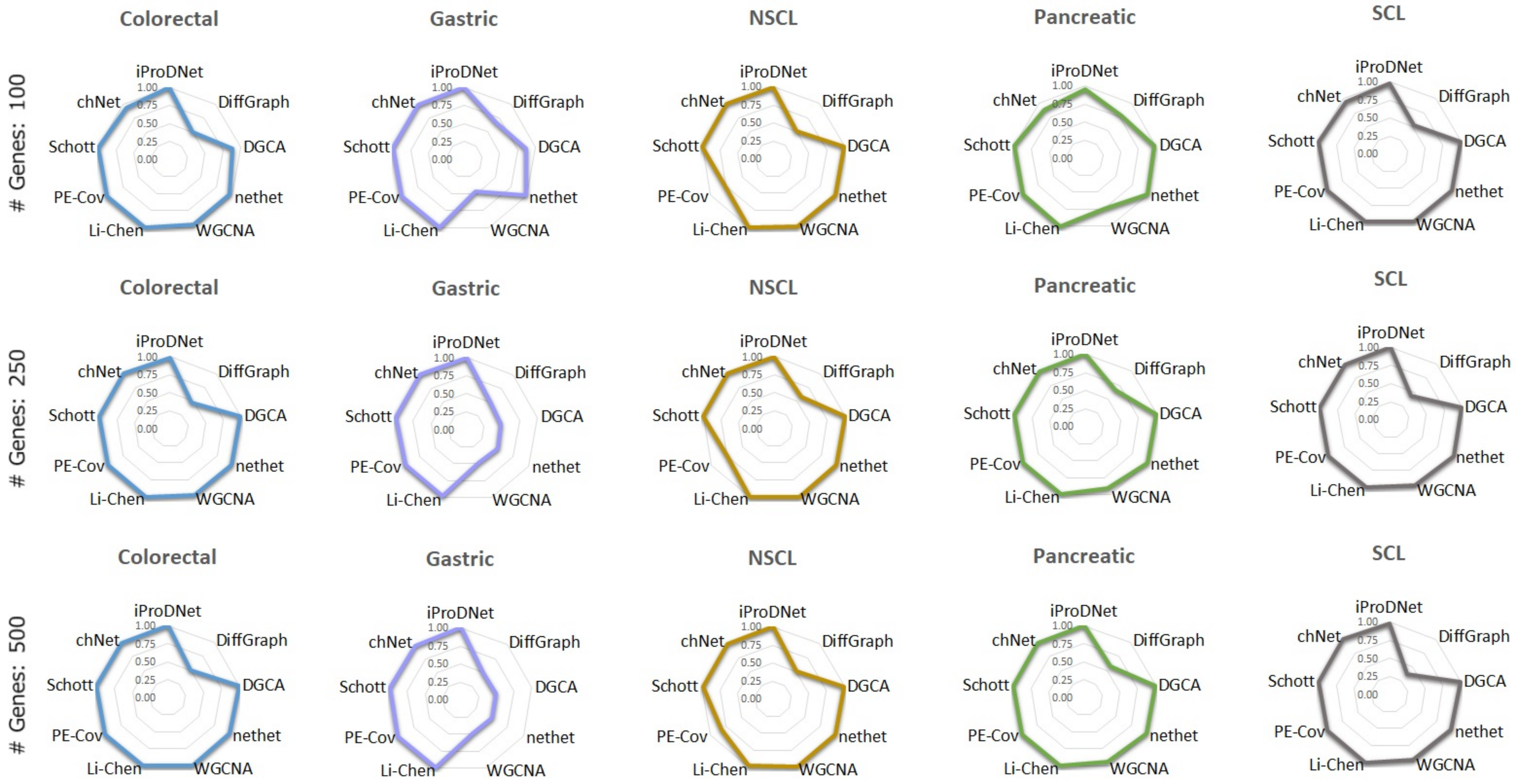
Colorectal
Gastric
NSCL
Pancreatic
SCL
Genes: 100
Genes: 250
Genes: 500
iProDNet
DiffGraph
DGCA
nethet
WGCNA
Li-Chen
PE-Cov
Schott
chNet
1.00
0.75
0.50
0.25
0.00
